# WCRF/AICR dietary adherence-associated metabolic bridge phenotypes and incident lung cancer risk: a prospective cohort study of the UK Biobank

**DOI:** 10.3389/fnut.2026.1877513

**Published:** 2026-07-13

**Authors:** Gaofeng Liang, Jianjian Ying, Jinxian He, Hongyan Yu, Tian Chen, Mengqiu Tang, Chengbin Lin, Liang Zhang, Weiyu Shen

**Affiliations:** 1 Department of Thoracic Surgery, The Affiliated Lihuili Hospital of Ningbo University, Ningbo, Zhejiang, China; 2 Department of Radiation Oncology, The Affiliated Lihuili Hospital of Ningbo University, Ningbo, Zhejiang, China; 3 Department of Respiratory Medicine, Ningbo Medical Center Lihuili Hospital, Ningbo, Zhejiang, China

**Keywords:** dietary adherence, lung cancer, metabolic signature, metabolomics, NMR spectroscopy, WCRF/AICR

## Abstract

**Background:**

Lung cancer is the leading cause of cancer-related mortality worldwide. Although the World Cancer Research Fund/American Institute for Cancer Research (WCRF/AICR) dietary recommendations are widely endorsed, their association with lung cancer risk and related circulating metabolic phenotypes remains unclear.

**Methods:**

We derived a UK Biobank-adapted WCRF/AICR dietary adherence score from six components. Among 91,917 participants with complete dietary, covariate, follow-up, and metabolomic data, we constructed a diet-related plasma metabolomic signature and an FDR-restricted bridge score. Cox and Fine-Gray models examined associations with incident lung cancer, with Benjamini–Hochberg correction applied to high-dimensional analyses.

**Results:**

Over follow-up, 470 incident lung cancer cases occurred. The dietary score was not significantly associated with lung cancer risk. In contrast, the metabolomic signature was associated with lower risk in both Cox (HR per 1-SD: 0.816, 95% CI: 0.752–0.885; *p* < 0.001) and Fine-Gray models (SHR: 0.830, 95% CI: 0.778–0.885; *p* < 0.001). The bridge score showed a borderline association in single-exposure Cox models (HR: 0.907, 95% CI: 0.823–1.000; *p* = 0.051) but was attenuated in joint models. Three plasma fatty acid measures met dual-path FDR criteria: saturated fatty acids (higher risk), linoleic acid, and polyunsaturated fatty acids (both lower risk). Sensitivity analyses excluding early events supported robustness.

**Conclusion:**

The questionnaire-based dietary score was not directly associated with lung cancer risk, whereas a plasma metabolomic signature showed a consistent inverse association. Fatty acid compositional measures may represent candidate metabolic biomarkers linking diet to lung cancer risk. These associative findings suggest that metabolic profiling may complement dietary questionnaires in future studies.

## 1 Introduction

Lung cancer is among the most prevalent and lethal malignancies globally, consistently ranking as the leading cause of cancer-related mortality (1). Beyond well-established risk factors such as tobacco smoking, air pollution, and genetic susceptibility, the persistently rising incidence among never-smokers and marked individual variability in smoking-associated lung cancer risk suggest that modifiable lifestyle factors—particularly dietary patterns—may play a more substantial yet underappreciated etiological role (2). However, the epidemiological literature on diet and lung cancer risk remains inconsistent and exhibits critical gaps. Some cohort and case–control studies have reported that dietary patterns characterized by high fruit and vegetable intake, high dietary fiber, and limited consumption of red and processed meat, sugar-sweetened beverages, and alcohol may be associated with reduced lung cancer risk (3, 4). Yet large-scale studies have found no significant independent association between conventional dietary scores and lung cancer incidence, with paradoxical findings even emerging in certain subpopulations, precluding reliable confirmation of a dietary protective effect against lung cancer (5).

The World Cancer Research Fund/American Institute for Cancer Research (WCRF/AICR) cancer prevention dietary recommendations, encompassing multiple dietary components (fruits and vegetables, dietary fiber, limited red/processed meat, limited sugar-sweetened beverages, limited alcohol, ultra-processed food intake), are considered a cornerstone of cancer prevention public health strategy. WCRF/AICR adherence score was created which aimed to standardise how adherence to these recommendations is assessed. Meta-analyses have indicated that greater adherence is associated with reduced risks of overall cancer, colorectal cancer, and breast cancer (6, 7). Previous studies have directly examined the association between the overall WCRF/AICR adherence score and lung cancer risk with inconsistent findings.

Critically, the WCRF/AICR dietary guidelines—as the preeminent cancer prevention dietary framework—have not been examined in relation to lung cancer risk through metabolic bridge pathways (8, 9). “Metabolite bridge pathways” refers to a composite score constructed from diet-associated metabolites ranked by their exploratory bridge index (the product of diet-metabolite and metabolite-cancer associations), designed to capture the metabolic consequences of dietary adherence. Metabolomics comprehensively captures diet-induced changes in circulating metabolites, serving as an ideal bridge between external dietary exposures and internal tumor susceptibility (10, 11). Recent studies have confirmed that metabolic modules encompassing fatty acid composition, lipoprotein lipids, amino acid profiles, and energy metabolism are closely linked to lung cancer development, and elevated saturated fatty acid proportions, reduced polyunsaturated fatty acid content, and disturbed energy metabolism may all participate in pulmonary carcinogenesis (12–15). Yet no study has systematically constructed WCRF/AICR diet-associated metabolic signatures using large-scale prospective cohort data, rigorously validated their independent predictive value for lung cancer risk, or elucidated their associative bridging role.

The existing literature is hampered by three major limitations (7, 16, 17): reliance on single or limited dietary assessments introduces large exposure measurement error; the biological pathways through which diet influences lung carcinogenesis remain largely unexplored; and the failure to integrate high-throughput metabolomics data has prevented the identification of quantifiable metabolic bridge phenotypes, limiting the biological explanatory power of dietary cancer prevention research. Accordingly, this study pursued three complementary objectives: (1) to examine whether WCRF/AICR dietary adherence independently predicts incident lung cancer risk after multivariable adjustment in a large prospective cohort; (2) to construct and validate diet-associated metabolomic signatures and metabolite scores as metabolic candidate biomarkers connecting dietary adherence to lung cancer risk; and (3) to evaluate the incremental predictive value of these metabolic phenotypes beyond conventional dietary scores and to characterize the key metabolic modules involved, with the aim of providing evidence-based targets for precision dietary lung cancer prevention.

## 2 Materials and methods

### 2.1 Study design and data sources

This study represents a prospective cohort analysis based on harmonized UK Biobank data (18), designed to investigate the associations among adherence to the WCRF/AICR cancer prevention dietary guidelines, associated metabolic phenotypes, and incident lung cancer risk. The UK Biobank is a large population-based prospective cohort that recruited approximately 500,000 community volunteers aged 40–69 years at 22 assessment centers across the United Kingdom between 2006 and 2010 (19). The project collected detailed baseline data including questionnaires, physical examinations, and multi-omics biological samples, with prospective follow-up of long-term health outcomes. The temporal sequence was as follows: NMR metabolomic blood samples were collected at the baseline assessment visit (2006–2010); 24-h dietary recalls were collected between 2011 and 2012; follow-up for incident lung cancer began at the date of each participant’s last valid dietary recall, ensuring that both dietary exposure and metabolomic measurements preceded outcome assessment. This design ensured that dietary exposure assessment preceded outcome ascertainment. Ethical approval was obtained from the North West Multi-centre Research Ethics Committee in the UK. All participants provided written informed consent at enrollment. The current analyses were carried out under Application Number 56312.

Multidimensional data sources included: (1) dietary exposure data derived from repeated 24-h dietary recalls completed online using the Oxford WebQ system, used to estimate food and nutrient intake and construct the UK Biobank-adapted WCRF/AICR dietary adherence score; (2) plasma metabolomics data from the UK Biobank NMR-based Nightingale Health platform, used to construct diet-related plasma metabolomic phenotypes; and (3) outcome and mortality data, with dates and diagnoses of incident lung cancer, other incident cancers, and death obtained through linkage to national electronic health records and cancer registries.

### 2.2 Study population and follow-up

A total of 210,722 participants had a WCRF/AICR dietary score record. Among them, 94,991 had at least one valid 24-h dietary recall and a non-missing final dietary assessment date. After excluding participants with any cancer diagnosed on or before the index date, 93,851 participants remained. Further restriction to participants with positive follow-up time, available NMR metabolomics, and complete primary-model covariates yielded a final analytic sample of 91,917 participants. The detailed participant flow is presented in Supplementary Table S1.

A valid dietary recall was defined as having a recall date, usable dietary or nutrient records, and the ability to calculate all WCRF/AICR dietary score components. The follow-up start date was fixed at each participant’s last valid 24-h dietary recall date to minimize time-reversed exposure–outcome relationships. Exclusion criteria included any cancer diagnosis on or before the follow-up start date, follow-up time ≤0, unavailable NMR metabolomic measurements, and missing values for the primary exposure or model covariates.

We evaluated the feasibility of broadening the dietary recall criterion to participants with at least one valid 24-h dietary recall. However, after requiring a complete WCRF/AICR dietary score, available NMR metabolomics, positive follow-up time, and complete covariate information, no additional participants beyond those with at least two valid recalls entered the final analytic sample. Therefore, the final analytic sample was equivalent to the sample restricted to participants with at least two valid dietary recalls.

### 2.3 UK biobank-adapted WCRF/AICR dietary adherence score

The UK Biobank-adapted WCRF/AICR dietary adherence score comprised six dietary components: fruit and vegetables, dietary fibre, an ultra-processed food/fast-food proxy, red and processed meat, sugar-sweetened beverages, and alcohol. The score is explicitly termed “UK Biobank-adapted” because the UPF/fast-food component was adapted for UK Biobank using cohort tertiles of the NOVA-UPF weight percentage; all other components used official or adapted thresholds from WebQ-derived estimates (20). Detailed scoring criteria, thresholds, and UK Biobank adaptations are provided in Supplementary Table S2. Fruit and vegetables and dietary fibre each contributed a maximum of 0.5 points, while the remaining four components each contributed a maximum of 1.0 point, yielding a total score range of 0–5 points. Higher scores indicated greater adherence to cancer prevention dietary recommendations. The academic names, operational definitions, and scoring rules for all dietary components are provided in Supplementary Table S2. The primary dietary exposure was the standardized UK Biobank-adapted WCRF/AICR dietary adherence score, entered as a continuous variable per 1-SD increment in Cox models. Quartile-based analyses were also conducted, with the lowest quartile used as the reference group.

### 2.4 Metabolomic signature and high-dimensional bridge analysis

Step 1: Diet-associated plasma metabolomic signature training. The metabolomic signature was constructed to capture the plasma metabolic phenotype associated with the UK Biobank-adapted WCRF/AICR dietary adherence score. The NMR metabolomics analysis included 251 Nightingale Health plasma metabolite measures. Metabolites with >20% missingness were excluded, and remaining missing values were imputed using the median in the training set. Metabolites were standardized using training-set means and standard deviations.

The analytical cohort was randomly split into training and test sets using an 80/20 split. To reduce dimensionality, the top 150 metabolites most correlated with the dietary score in the training set were pre-selected. Elastic net regression with *α* = 0.5 was then applied using five-fold cross-validation. This procedure selected 27 plasma metabolite measures. Pearson correlations between observed dietary scores and predicted signatures were 0.262 in both the training and test sets, indicating similar performance across the two datasets.

Step 2: Exploratory high-dimensional bridge association analysis. For each of the 251 plasma metabolites measured by the Nightingale Health NMR platform, two directed associations were estimated. The “a path” represented the association between the UK Biobank-adapted WCRF/AICR dietary score and each plasma metabolite, estimated using covariate-adjusted linear regression. The “b path” represented the association between each plasma metabolite and incident lung cancer, estimated using Cox proportional hazards models simultaneously adjusted for the dietary score and covariates.

Because 251 metabolites were tested, Benjamini–Hochberg false discovery rate correction was applied separately across all 251 a-path tests and all 251 b-path tests. The a × b product was used as an exploratory bridge index for ranking and visualization only. No formal mediated proportion was estimated, and all bridge analyses were interpreted as exploratory and hypothesis-generating rather than confirmatory mediation analyses.

Step 3: FDR-restricted plasma bridge score construction. The plasma bridge score was constructed only from metabolites that met FDR < 0.05 in both the diet-to-metabolite path and the metabolite-to-lung cancer path. Three plasma fatty acid proportional measures met these dual-path FDR criteria: saturated fatty acids to total fatty acids percentage, linoleic acid to total fatty acids percentage, and polyunsaturated fatty acids to total fatty acids percentage.

For each selected metabolite, a direction-adjusted weight of −a × b was used so that higher bridge scores reflected a more favourable diet-related plasma metabolic phenotype. The directionality logic is as follows: for a protective metabolite such as linoleic acid, higher dietary adherence is associated with higher metabolite levels (a > 0), and higher levels are associated with lower cancer risk (b < 0), yielding –a × b > 0. Conversely, for a risk-related metabolite such as SFA percentage, higher dietary adherence is associated with lower levels (a < 0), and higher levels are associated with higher cancer risk (b > 0), also yielding –a × b > 0. Thus, regardless of whether a metabolite is positively or negatively associated with diet, the weighting scheme ensures that all selected metabolites contribute positively to the bridge score, such that higher scores always reflect a more favorable metabolic phenotype. The weighted score was then standardized to mean 0 and SD 1 for model entry. Linoleic acid is an omega-6 polyunsaturated fatty acid; therefore, the selected fatty acid proportional measures were interpreted as correlated and hierarchically related plasma lipid composition measures rather than independent biological pathways. The dual-path FDR-confirmed metabolites are listed in Supplementary Table S3.

### 2.5 Outcome definition

The primary outcome was incident lung cancer, defined by ICD-10 codes C33–C34, with events defined as diagnoses occurring after the follow-up start date. Participants were censored at the first occurrence of a non-lung cancer primary cancer diagnosis, death, or the administrative end-of-follow-up date (1 July 2025). In Fine–Gray competing risk models, first incident non-lung cancer and death were treated as competing events.

### 2.6 Covariates

Model covariates were pre-specified to include demographic, lifestyle, metabolic health, and dietary assessment intensity variables. Continuous covariates included age at enrollment (years), BMI (kg/m^2^), physical activity (log-transformed total MET-min/week), total energy intake (mean, standardized per 1-SD), and number of valid 24-h dietary recalls. Categorical covariates included sex (female/male); ethnicity (White vs. non-White or unknown); household income (low, middle, high, unknown); smoking status (never, previous, current, unknown; four levels retained to avoid over-condensation of residual confounding); alcohol use (current vs. non-current or unknown); and diabetes status (no vs. yes or unknown). The details are listed in Supplementary Table S4.

### 2.7 Statistical analysis

Cox proportional hazards regression was used to estimate associations of the UK Biobank-adapted WCRF/AICR dietary adherence score, diet-related plasma metabolomic signature, and FDR-restricted plasma bridge score with incident lung cancer risk. All continuous exposures were standardized and analysed per 1-SD increment. Effect estimates are presented as hazard ratios (HRs) with 95% confidence intervals (CIs).

Primary models evaluated the WCRF/AICR dietary score, the plasma metabolomic signature, and the FDR-restricted plasma bridge score in single-exposure models. The dietary score was additionally analysed across quartiles, with the lowest quartile as the reference and a trend test performed across quartiles. Joint models then included the dietary score, metabolomic signature, and bridge score simultaneously to assess whether the bridge score contributed information independent of the broader diet-related plasma metabolomic signature.

To assess potential reverse causality from subclinical lung cancer, sensitivity analyses excluded lung cancer events occurring within the first 2, 3, and 5 years of follow-up. Fine–Gray competing risk models were used to account for first incident non-lung cancer and death as competing events. Selection bias related to the requirement for complete dietary, covariate, and NMR metabolomic data was evaluated using inverse probability weighting. The selection model used inclusion in the final NMR analytic sample as the outcome and included age, sex, ethnicity, educational attainment, household income, smoking status, alcohol consumption, body mass index, physical activity, diabetes status, total energy intake, WCRF/AICR dietary adherence score, and number of valid 24-h dietary recalls. Stabilized weights were truncated at the 1st and 99th percentiles.

Baseline characteristics were compared across quartiles of the dietary adherence score. Included and excluded eligible participants were also compared using standardized mean differences to assess potential selection bias. Module-level and joint diet–metabolic analyses were treated as exploratory because of the limited number of incident lung cancer cases and multiple testing burden. Benjamini–Hochberg FDR correction was applied to the high-dimensional a-path and b-path metabolite analyses. All analyses were performed in R, and two-sided *p* values <0.05 were considered statistically significant unless FDR correction was specified.

## 3 Results

### 3.1 Baseline characteristics

The final analytic cohort comprised 91,917 participants with complete WCRF/AICR dietary score data, positive follow-up time, available NMR-based plasma metabolomics, and complete primary-model covariates. During follow-up, 470 incident lung cancer cases were identified. The detailed participant selection process is shown in Supplementary Table S1.

Baseline characteristics according to quartiles of the UK Biobank-adapted WCRF/AICR dietary adherence score are presented in Table 1. Across increasing quartiles of the dietary adherence score, the proportion of women increased from 44.2% in Q1 to 62.6% in Q4, mean body mass index decreased from 27.30 kg/m^2^ to 25.91 kg/m^2^, and the proportion of current smokers decreased from 8.5 to 5.1%. Physical activity was higher across increasing dietary score quartiles, whereas total energy intake decreased. These differences suggest that participants with higher WCRF/AICR dietary adherence generally had more favourable lifestyle profiles.

**Table 1:** Baseline characteristics by quartiles of the WCRF/AICR dietary adherence score.

Characteristic	Category	Q1	Q2	Q3	Q4	*P* value
Age, years		54.72 (8.01)	55.54 (7.87)	56.05 (7.77)	55.89 (7.70)	<0.001
Body mass index, kg/m^2^		27.30 (4.62)	26.76 (4.41)	26.48 (4.42)	25.91 (4.46)	<0.001
Physical activity, log MET-min/week		7.16 (1.45)	7.20 (1.39)	7.31 (1.28)	7.36 (1.28)	<0.001
Total energy intake		9325.27 (2265.33)	8839.43 (2178.28)	8464.61 (2033.92)	8102.45 (2054.19)	<0.001
WCRF/AICR dietary score		1.29 (0.42)	2.26 (0.20)	2.99 (0.21)	3.93 (0.38)	<0.001
Valid dietary recalls, *n*		3.06 (0.90)	2.99 (0.90)	2.94 (0.90)	2.82 (0.87)	<0.001
Sex	Female	10,586 (44.2%)	12,049 (49.0%)	13,334 (57.5%)	12,623 (62.6%)	<0.001
	Male	13,363 (55.8%)	12,563 (51.0%)	9,869 (42.5%)	7,530 (37.4%)	
Ethnicity	White	23,329 (97.4%)	23,874 (97.0%)	22,506 (97.0%)	19,284 (95.7%)	<0.001
	Non-white or unknown	620 (2.6%)	738 (3.0%)	697 (3.0%)	869 (4.3%)	
Education	University degree	10,687 (44.6%)	11,882 (48.3%)	11,783 (50.8%)	11,001 (54.6%)	<0.001
	Other qualification	11,848 (49.5%)	11,309 (45.9%)	10,166 (43.8%)	8,135 (40.4%)	
	No formal qualification	1,371 (5.7%)	1,363 (5.5%)	1,212 (5.2%)	973 (4.8%)	
	Unknown	43 (0.2%)	58 (0.2%)	42 (0.2%)	44 (0.2%)	
Household income	Low	7,644 (31.9%)	8,021 (32.6%)	7,448 (32.1%)	6,806 (33.8%)	<0.001
	Middle	6,669 (27.8%)	6,618 (26.9%)	6,196 (26.7%)	5,282 (26.2%)	
	High	7,974 (33.3%)	8,169 (33.2%)	7,808 (33.7%)	6,468 (32.1%)	
	Unknown	1,662 (6.9%)	1,804 (7.3%)	1,751 (7.5%)	1,597 (7.9%)	
Smoking status	Never	13,428 (56.1%)	14,000 (56.9%)	13,305 (57.3%)	12,128 (60.2%)	<0.001
	Previous	8,444 (35.3%)	8,706 (35.4%)	8,359 (36.0%)	6,957 (34.5%)	
	Current	2,036 (8.5%)	1,876 (7.6%)	1,508 (6.5%)	1,036 (5.1%)	
	Unknown	41 (0.2%)	30 (0.1%)	31 (0.1%)	32 (0.2%)	
Alcohol use	Current	22,991 (96.0%)	23,430 (95.2%)	21,944 (94.6%)	18,477 (91.7%)	<0.001
	Non-current or unknown	958 (4.0%)	1,182 (4.8%)	1,259 (5.4%)	1,676 (8.3%)	
Doctor-diagnosed diabetes	No or unknown	23,149 (96.7%)	23,646 (96.1%)	22,337 (96.3%)	19,427 (96.4%)	0.006
	Yes	800 (3.3%)	966 (3.9%)	866 (3.7%)	726 (3.6%)	

Continuous variables are mean (SD); categorical variables are *n* (%). *P* values were calculated using Kruskal–Wallis tests for continuous variables and chi-square tests for categorical variables.

Compared with eligible participants who were not included in the final analytic sample, included participants were slightly younger, had lower BMI, higher total energy intake, and were more likely to have a university degree and higher household income. However, most standardized mean differences were small; the largest absolute standardized differences were observed for BMI and total energy intake. The full comparison between included and excluded participants is shown in Supplementary Table S5.

### 3.2 Distribution of UK Biobank-adapted WCRF/AICR dietary score and metabolomic signature

The UK Biobank-adapted WCRF/AICR dietary adherence score ranged from 0 to 5 points. In the final analytic cohort, the mean score was 2.56 (SD: 1.00), with quartile-specific means of 1.29, 2.26, 2.99, and 3.93 from Q1 to Q4, respectively. The scoring components and operational definitions are provided in Supplementary Table S2.

The diet-related plasma metabolomic signature was trained using 251 Nightingale Health NMR-derived plasma metabolite measures. After correlation pre-screening and elastic net regression, 27 plasma metabolite measures were retained. Pearson correlations between observed dietary scores and predicted metabolomic signatures were 0.262 in both the training and test sets, indicating comparable performance across the two datasets.

### 3.3 Associations of UK Biobank-adapted WCRF/AICR dietary score, metabolomic signature, and bridge score with incident lung cancer risk

In multivariable Cox models, the UK Biobank-adapted WCRF/AICR dietary adherence score was not significantly associated with incident lung cancer risk. The HR per 1-SD increment was 0.989 (95% CI: 0.898–1.089; *p* = 0.821). Quartile-based analyses also showed no clear dose–response association: compared with Q1, the HR for Q4 was 1.004 (95% CI: 0.759–1.328; *p* = 0.978), with *P* for trend = 0.924.

In contrast, the diet-related plasma metabolomic signature was associated with lower incident lung cancer risk. The HR per 1-SD increment was 0.816 (95% CI: 0.752–0.885; *p* < 0.001) in the single-exposure model. The FDR-restricted plasma bridge score showed a borderline inverse association with lung cancer risk, with an HR of 0.907 (95% CI: 0.823–1.000; *p* = 0.051).

When the dietary score, plasma metabolomic signature, and FDR-restricted plasma bridge score were included simultaneously, the metabolomic signature remained associated with lower lung cancer risk (HR: 0.822, 95% CI: 0.754–0.896; *p* < 0.001), whereas the bridge score was attenuated and no longer statistically significant (HR: 0.949, 95% CI: 0.858–1.049; *p* = 0.305). In Fine–Gray competing-risk models, the plasma metabolomic signature remained associated with lower lung cancer risk (SHR: 0.830, 95% CI: 0.778–0.885; *p* < 0.001), while the bridge score did not reach statistical significance (SHR: 0.912, 95% CI: 0.824–1.010; *p* = 0.077). These findings indicate that the primary risk-related information was captured by the broader diet-related plasma metabolomic signature, while the FDR-restricted bridge score did not provide clear independent information beyond the signature. The main association results are shown in Table 2.

**Table 2:** WCRF/AICR dietary adherence, plasma metabolomic profiles, and incident lung cancer.

Exposure	Contrast	Model	Participants	Cases	HR/SHR (95% CI)	*P* value
WCRF/AICR dietary score	Per 1-SD increment	Single-exposure	91,917	470	0.989 (0.898, 1.089)	0.821
WCRF/AICR dietary score	Q2 vs. Q1	Quartile model	91,917	470	1.023 (0.798, 1.312)	0.857
WCRF/AICR dietary score	Q3 vs. Q1	Quartile model	91,917	470	1.041 (0.806, 1.345)	0.756
WCRF/AICR dietary score	Q4 vs. Q1	Quartile model	91,917	470	1.004 (0.759, 1.328)	0.978
WCRF/AICR dietary score	P for trend	Quartile model	91,917	470		0.924
Diet-related plasma metabolomic signature	Per 1-SD increment	Single-exposure	91,917	470	0.816 (0.752, 0.885)	<0.001
FDR-restricted plasma bridge score	Per 1-SD increment	Single-exposure	91,917	470	0.907 (0.823, 1.000)	0.051
Diet-related plasma metabolomic signature	Per 1-SD increment	Diet + signature + bridge	91,917	470	0.822 (0.754, 0.896)	<0.001
FDR-restricted plasma bridge score	Per 1-SD increment	Diet + signature + bridge	91,917	470	0.949 (0.858, 1.049)	0.305
Fine-Gray competing risk	-	WCRF/AICR score	91,917	470	0.992 (0.897, 1.096)	0.871
Fine-Gray competing risk	-	Plasma metabolomic signature	91,917	470	0.830 (0.778, 0.885)	<0.001
Fine-Gray competing risk	-	Plasma bridge score	91,917	470	0.912 (0.824, 1.010)	0.077

Models adjusted for age, sex, ethnicity, education, household income, smoking status, alcohol use, BMI, physical activity, diabetes, total energy intake, and number of valid dietary recalls.

### 3.4 Exploratory high-dimensional bridge metabolite analysis

In the high-dimensional bridge analysis, 251 diet-to-metabolite a-path tests and 251 metabolite-to-lung cancer b-path tests were corrected separately using the Benjamini–Hochberg false discovery rate procedure. Among the 251 b-path tests, 18 plasma metabolite measures had nominal *p* values <0.05; after FDR correction, three plasma fatty acid proportional measures remained significant at FDR < 0.05 and also met the a-path FDR criterion.

The three dual-path FDR-confirmed plasma metabolites were saturated fatty acids to total fatty acids percentage, linoleic acid to total fatty acids percentage, and polyunsaturated fatty acids to total fatty acids percentage. Higher UK Biobank-adapted WCRF/AICR dietary adherence was associated with lower plasma saturated fatty acid proportion and higher plasma linoleic acid and polyunsaturated fatty acid proportions.

**Figure 1:**
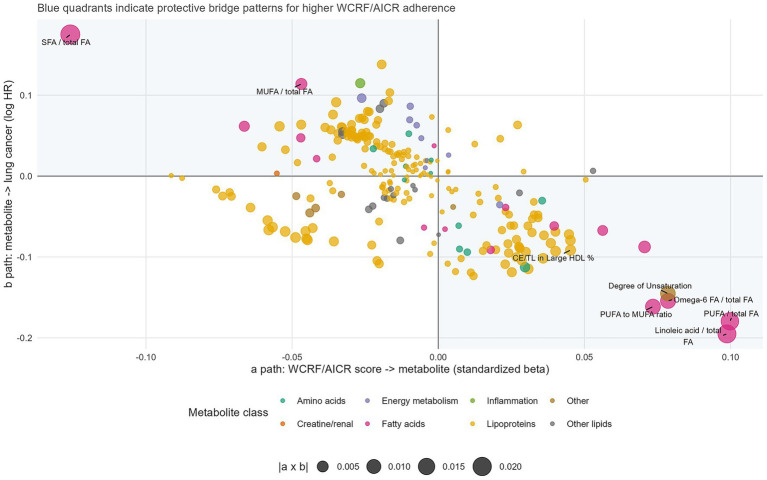
Exploratory high-dimensional bridge landscape. Each bubble represents one of 251 Nightingale NMR-derived plasma metabolite measures. The x-axis shows the a-path coefficient for the association between the UK Biobank-adapted WCRF/AICR dietary adherence score and each plasma metabolite. The y-axis shows the b-path log hazard ratio for the association between each plasma metabolite and incident lung cancer, adjusted for the WCRF/AICR dietary score and model covariates. Bubble size reflects the absolute a × b bridge index. Black outlines identify metabolites meeting Benjamini–Hochberg false discovery rate (BH-FDR) < 0.05 in both the diet-to-metabolite and metabolite-to-lung cancer paths. Labeled metabolites represent the main dual-path FDR-confirmed plasma fatty acid proportional measures.

**Figure 2:**
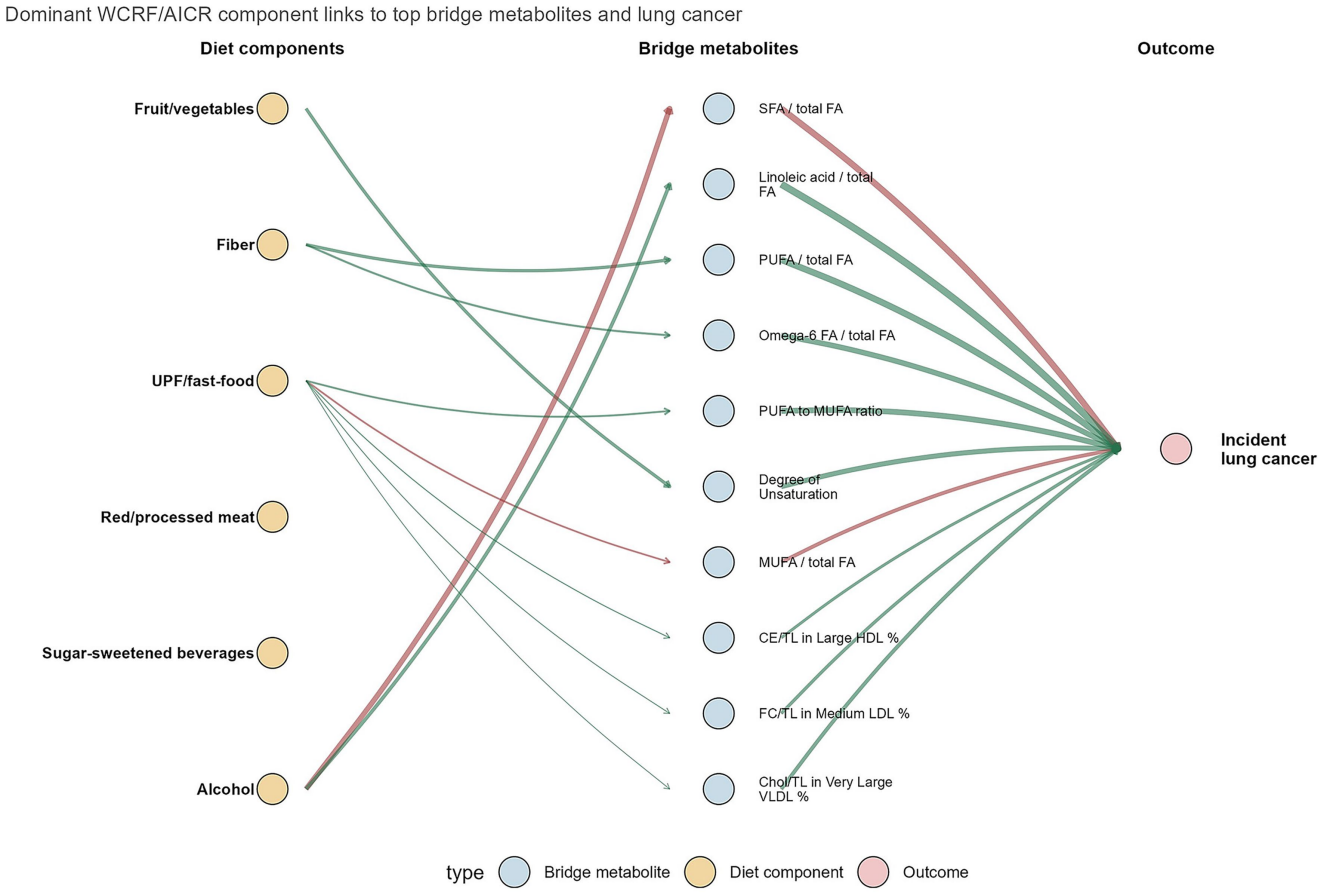
Plasma fatty acid proportional measures meeting dual-path FDR criteria. Hazard ratios and 95% confidence intervals correspond to a 1-SD higher Nightingale NMR-derived plasma measure. Saturated fatty acids to total fatty acids percentage was associated with higher incident lung cancer risk, whereas linoleic acid to total fatty acids percentage and polyunsaturated fatty acids to total fatty acids percentage were associated with lower risk. Linoleic acid is an omega-6 polyunsaturated fatty acid; therefore, the displayed measures are correlated and hierarchically related rather than independent biological pathways.

For the b path, each 1-SD higher plasma saturated fatty acids to total fatty acids percentage was associated with higher lung cancer risk (HR: 1.193, 95% CI: 1.095–1.300; FDR = 0.009). Conversely, higher plasma linoleic acid to total fatty acids percentage and higher plasma polyunsaturated fatty acids to total fatty acids percentage were associated with lower risk, with HRs of 0.826 (95% CI: 0.752–0.908; FDR = 0.009) and 0.838 (95% CI: 0.765–0.919; FDR = 0.014), respectively. Because linoleic acid is an omega-6 polyunsaturated fatty acid, these fatty acid proportional measures are correlated and hierarchically related, and should not be interpreted as independent biological pathways. These exploratory findings are displayed in Figures 1, 2 and detailed metabolite-level results are shown in Supplementary Table S3.

### 3.5 Exploratory metabolic modules and joint diet–metabolic risk stratification

Exploratory module-level analyses suggested that several plasma metabolite modules were associated with incident lung cancer risk. The fatty acid profile module was inversely associated with lung cancer risk (HR per 1-SD increment: 0.866, 95% CI: 0.785–0.956; *p* = 0.004; FDR = 0.008). Other metabolic signals were also inversely associated with risk (HR: 0.893, 95% CI: 0.812–0.982; *p* = 0.019; FDR = 0.025). The lipoprotein-lipid profile showed a borderline inverse association (HR: 0.913, 95% CI: 0.831–1.003; *p* = 0.059; FDR = 0.059), whereas the energy metabolism module was positively associated with lung cancer risk (HR: 1.141, 95% CI: 1.052–1.238; *p* = 0.001; FDR = 0.006). These module-level findings are presented as exploratory signal summaries because the modules were derived and tested in the same dataset (Supplementary Table S6).

**Figure 3:**
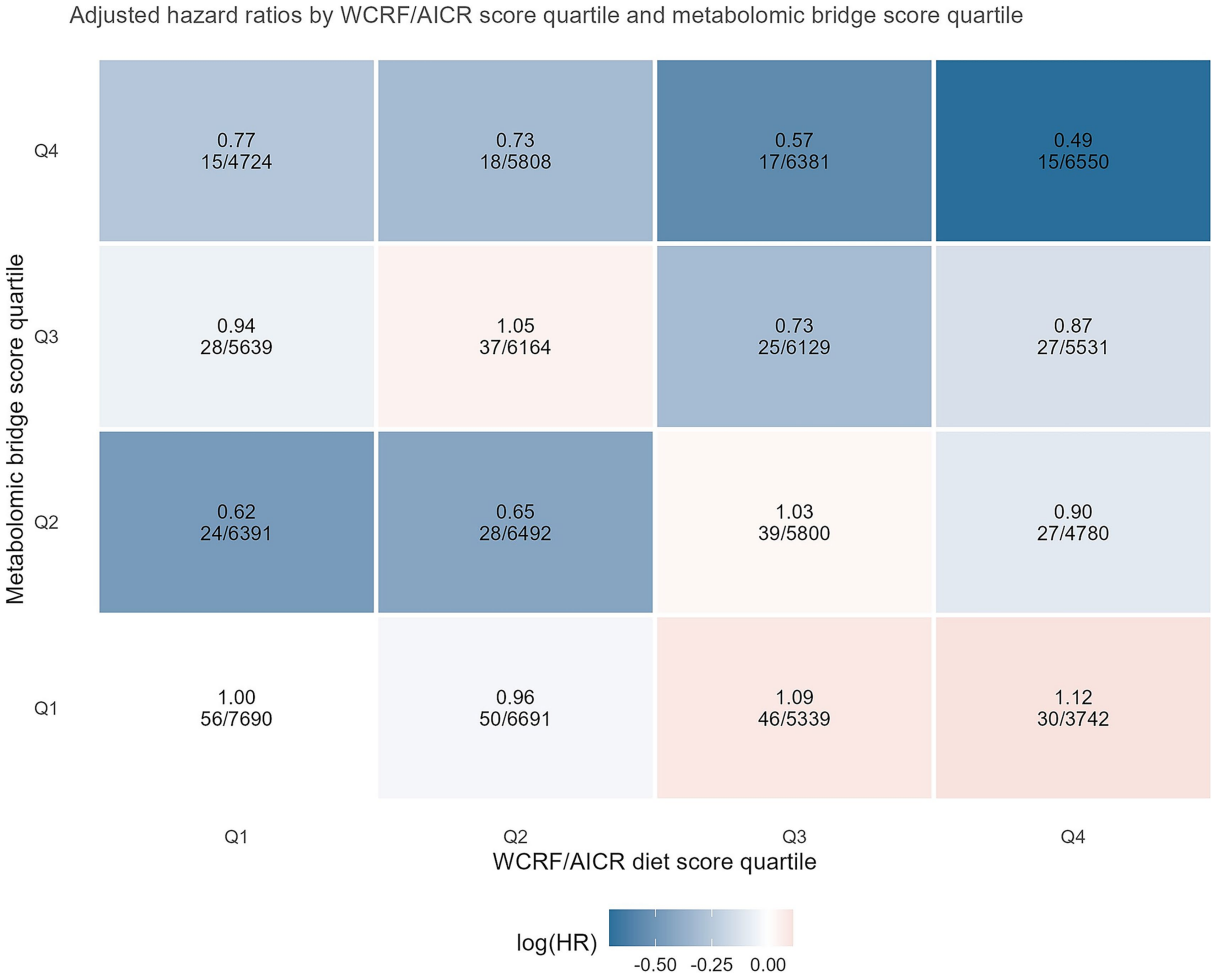
Exploratory joint analysis of WCRF/AICR dietary score and plasma metabolomic bridge score categories. Participants were cross-classified into 16 mutually exclusive categories according to quartiles of the UK Biobank-adapted WCRF/AICR dietary adherence score and quartiles of the FDR-restricted plasma bridge score. Hazard ratios and 95% confidence intervals are shown relative to the reference group of diet Q1 and bridge score Q1. The joint categories are displayed without imposing a continuous trend. Because confidence intervals were wide and no clear monotonic gradient was observed, this joint analysis should be interpreted as exploratory and hypothesis-generating.

The exploratory joint analysis of WCRF/AICR dietary score quartiles and plasma bridge score quartiles did not show a clear monotonic risk gradient. Using the joint category of diet Q1 and bridge score Q1 as the reference, participants in the diet Q4 and bridge score Q4 category had an HR of 0.848 (95% CI: 0.487–1.477; *p* = 0.560). The wide confidence intervals and lack of a clear gradient indicate that the joint stratification results should be interpreted cautiously and regarded as hypothesis-generating. The revised joint analysis is shown in Figure 3.

### 3.6 Sensitivity, selection-weighted, and competing-risk analyses

Sensitivity analyses excluding early lung cancer events supported the robustness of the plasma metabolomic signature findings. After excluding lung cancer events occurring within the first 2 years of follow-up, the HR for the plasma metabolomic signature was 0.802 (95% CI: 0.737–0.873; *p* < 0.001). The corresponding HRs were 0.800 (95% CI: 0.729–0.879; p < 0.001) after excluding events within the first 3 years and 0.799 (95% CI: 0.718–0.889; *p* < 0.001) after excluding events within the first 5 years.

By contrast, the FDR-restricted plasma bridge score was not statistically significant in the lagged sensitivity analyses. The HRs were 0.947 (95% CI: 0.850–1.055; *p* = 0.323) after excluding events within the first 2 years, 0.951 (95% CI: 0.847–1.068; *p* = 0.398) after excluding events within the first 3 years, and 0.927 (95% CI: 0.813–1.057; *p* = 0.260) after excluding events within the first 5 years.

Selection-weighted analyses yielded results consistent with the primary findings. In the selection-IPW Cox model, the plasma metabolomic signature remained inversely associated with incident lung cancer risk (HR: 0.816, 95% CI: 0.763–0.873; *p* < 0.001), whereas the bridge score showed a non-significant inverse association (HR: 0.908, 95% CI: 0.819–1.005; *p* = 0.064). Selection-IPW specification and weight distribution was shown in Supplementary Table S7. These analyses are summarized in Supplementary Table S8.

## 4 Discussion

This study presents the first systematic investigation of the associations among UK Biobank-adapted WCRF/AICR cancer prevention dietary adherence, diet-associated circulating metabolic profiles, and incident lung cancer risk in a large-scale prospective cohort of 91,917 UK Biobank participants. The main findings were as follows. First, the questionnaire-based UK Biobank-adapted WCRF/AICR dietary adherence score was not significantly associated with incident lung cancer risk after multivariable adjustment. Second, the diet-related plasma metabolomic signature showed a consistent inverse association with lung cancer risk. Third, the FDR-restricted plasma bridge score showed only a borderline inverse association in the single-exposure model and was attenuated after simultaneous adjustment for the dietary score and metabolomic signature. Fourth, high-dimensional bridge analysis identified three plasma fatty acid proportional measures meeting dual-path FDR criteria, and module-level and joint diet–metabolic analyses should be interpreted as exploratory. These findings are consistent with the hypothesis that WCRF/AICR-adherent dietary patterns may be associated with lower lung cancer risk through remodeling of circulating metabolic profiles, whereas the dietary score alone showed no significant direct association in this cohort.

The absence of a statistically significant direct association between the UK Biobank-adapted WCRF/AICR dietary adherence score and incident lung cancer risk is consistent with findings from prior large-scale prospective studies (5). This null finding may partly reflect inherent measurement error in questionnaire-based dietary assessment, which can attenuate associations with outcomes that have long latency periods and multifactorial etiologies such as lung cancer. However, it should not be interpreted as evidence that dietary patterns have no relevance to lung cancer risk. Rather, it suggests that a questionnaire-based composite dietary score may not fully capture the biological heterogeneity of diet-related exposures in this setting. Nevertheless, it is important to note that metabolomic profiles are also influenced by genetic, metabolic, and other non-dietary factors, and our observational design cannot establish causality. Therefore, the stronger association observed for the plasma metabolomic signature should be interpreted as associative evidence rather than proof that circulating metabolites mediate dietary protection.

The core exploratory bridge signals centered on plasma fatty acid compositional profiles. Three plasma fatty acid proportional measures met FDR criteria in both the diet-to-metabolite and metabolite-to-lung cancer paths: saturated fatty acids to total fatty acids percentage, linoleic acid to total fatty acids percentage, and polyunsaturated fatty acids to total fatty acids percentage. Higher WCRF/AICR dietary adherence was associated with lower plasma saturated fatty acid proportion and higher plasma linoleic acid and polyunsaturated fatty acid proportions. In the b-path analysis, higher plasma saturated fatty acid proportion was associated with higher lung cancer risk, whereas higher plasma linoleic acid and polyunsaturated fatty acid proportions were associated with lower risk. Because linoleic acid is an omega-6 polyunsaturated fatty acid, these fatty acid proportional measures are correlated and hierarchically related and should not be interpreted as independent biological pathways. Previous experimental and epidemiological studies have suggested that fatty acid metabolism may be involved in inflammation, oxidative stress, immune regulation, and cancer-related metabolic reprogramming (20–27). However, the mechanistic relevance of these pathways to the present observational findings remains speculative, and the identified plasma fatty acid measures may also reflect broader dietary, metabolic, or behavioural profiles rather than direct causal intermediates. We acknowledge that omega-3 fatty acids did not meet the dual-path FDR significance threshold in our high-dimensional bridge analysis, which may reflect limited statistical power given the number of incident cases (*n* = 470) and the dominant signals from linoleic acid and total PUFA measures in our analytic framework. Future studies with larger case numbers are needed to clarify the role of omega-3 fatty acids in diet–lung cancer associations.

Exploratory module-level analyses provided additional context for the individual metabolite findings. The fatty acid profile module was inversely associated with lung cancer risk, and other metabolic signals also showed an inverse association. The lipoprotein-lipid profile showed a borderline inverse association, whereas the energy metabolism module was positively associated with lung cancer risk. These findings suggest that diet-related metabolic phenotypes may involve multiple interconnected metabolic domains. However, because the modules were derived and tested in the same dataset and the number of incident lung cancer events was limited, these module-level findings should be regarded as exploratory signal summaries rather than confirmatory pathway evidence.

The exploratory joint analysis of WCRF/AICR dietary score quartiles and plasma bridge score quartiles did not show a clear monotonic risk gradient. Participants in the highest dietary score and highest bridge score category did not have a statistically significant reduction in lung cancer risk compared with those in the lowest categories. Therefore, this joint analysis does not support a strong risk-stratification conclusion and should be interpreted cautiously as hypothesis-generating. Future studies with larger numbers of lung cancer events and external validation cohorts are needed to determine whether joint diet–metabolic phenotyping can improve etiological understanding or risk classification.

While the full NMR metabolomic platform is not feasible for routine clinical use, the top-ranked fatty acid ratio biomarkers identified in this study—particularly SFA% and LA%—could potentially be measured using targeted assays such as gas chromatography or mass spectrometry in clinical or research settings. Future research should evaluate whether a simplified panel of 3–5 fatty acid metrics retains predictive performance comparable to the full metabolomic signature. In addition, our findings may inform refinements to the conventional WCRF/AICR dietary score by incorporating circulating metabolic biomarkers, although prospective intervention studies would be required to validate whether such metabolomic-informed modifications improve predictive performance for cancer risk.

This study has several strengths. First, the prospective design and linkage to national cancer registry data reduced recall bias and improved outcome ascertainment. Second, repeated 24-h dietary recalls allowed more detailed dietary assessment than a single baseline food-frequency measure. Third, the availability of large-scale NMR-based plasma metabolomics enabled systematic evaluation of diet-related circulating metabolic phenotypes. Fourth, high-dimensional a-path and b-path analyses were corrected using Benjamini–Hochberg FDR procedures, reducing the likelihood of false-positive interpretation. Fifth, sensitivity analyses excluding lung cancer events within the first 2, 3, and 5 years, selection-IPW analyses, and Fine–Gray competing-risk models supported the robustness of the plasma metabolomic signature findings.

Several limitations deserve acknowledgment. First, despite adjustment for smoking status, residual confounding from unmeasured smoking characteristics, such as smoking intensity, duration, pack-years, and time since cessation, cannot be fully excluded. Second, individual-level long-term air pollution exposure data were not available for all participants in this analysis; air pollution is an established risk factor for lung cancer that may confound or modify diet–cancer associations. Third, the requirement for complete dietary score, covariate, and plasma metabolomic data may have introduced selection bias. Although included and excluded participants were compared and selection-IPW analyses were performed, residual selection bias from unmeasured factors cannot be ruled out. Fourth, Townsend deprivation index and assessment centre were not available in the current analytical export; socioeconomic position was adjusted using education and household income, but residual socioeconomic confounding may remain. Fifth, the study population was predominantly White British, limiting generalizability to Asian, African, and other populations with different dietary patterns, metabolic profiles, and lung cancer risk factor distributions. Sixth, despite using repeated dietary recalls, residual measurement error and long-term dietary changes during follow-up could not be fully captured. Seventh, as an observational cohort study, the design can identify associations but cannot establish causality; whether the identified metabolic phenotypes are causal intermediates or markers of unmeasured biological processes requires further investigation. Eighth, metabolomics assessment was restricted to the NMR-based Nightingale Health platform and did not include untargeted mass spectrometry, detailed lipidomics, or other small-molecule platforms. Ninth, the metabolomic signature and bridge score were derived and evaluated within the same overall cohort framework, and external validation was not available. In addition, the metabolomic signature and bridge score were derived specifically for lung cancer; their association with other cancers (e.g., colorectal, breast) remains unknown and requires separate investigation, as metabolic pathways may differ by cancer type. Tenth, the C-index improvement was modest (0.0036); external validation is needed before clinical application. DCA/NRI/IDI were not performed as our study was not a prediction model development. The bridge score, module-level, and joint analyses were exploratory only.

Future research should prioritize external validation in independent and multi-ethnic cohorts, prospective dietary intervention studies with repeated metabolomic profiling, and mechanistic studies to clarify whether plasma fatty acid compositional phenotypes are causal intermediates, biomarkers of broader dietary and metabolic states, or correlates of other lung cancer risk factors. Further work is also needed to determine whether simplified targeted panels of plasma fatty acid measures can reproducibly capture diet-related metabolic phenotypes relevant to lung cancer risk.

## Data Availability

The original contributions presented in the study are included in the article/Supplementary material, further inquiries can be directed to the corresponding author.
