# Supplementary material for: WCRF/AICR dietary adherence-associated metabolic bridge phenotypes and incident lung cancer risk: a prospective cohort study of the UK Biobank
Source: Front Nutr. 2026 Jul 13;13:1877513. doi: 10.3389/fnut.2026.1877513 (PMC13402149; doi:10.3389/fnut.2026.1877513)
Supplement: Supplementary file 1 [file Table_1.docx]

**Supplementary Material**

**Supplementary Table S1. Participant flow**

| **Selection step** | **Participants, n** |
| --- | --- |
| Participants with a WCRF/AICR score record | 210,722 |
| At least one valid 24-hour dietary recall | 94,991 |
| Non-missing final dietary assessment date | 94,991 |
| No cancer diagnosed on or before index date | 93,851 |
| Positive follow-up time | 93,851 |
| NMR metabolomics overlap | 91,917 |
| Complete primary-model covariates | 91,917 |

Note. The primary analysis required a complete WCRF/AICR score, positive follow-up, NMR metabolomics, and complete model covariates.

**Supplementary Table S2. WCRF/AICR dietary adherence score components**

| **Dietary component** | **Operational intake measure** | **Scoring approach** |
| --- | --- | --- |
| Fruit and vegetables | Daily combined intake | 0, 0.25, or 0.5 points according to <200, 200-<400, or >=400 g/day |
| Dietary fibre | Daily intake | 0, 0.25, or 0.5 points according to <15, 15-<30, or >=30 g/day |
| Ultra-processed food/fast-food proxy | Percentage of total food weight | 1, 0.5, or 0 points across ascending cohort tertiles |
| Red and processed meat | Weekly intake | Higher points for red meat <=500 g/week and lower processed meat intake |
| Sugar-sweetened beverages | Daily intake | 1 point for none, 0.5 for >0-250 g/day, and 0 for >250 g/day |
| Alcohol | Daily intake | Sex-specific scoring based on WCRF/AICR limits |

Note. The adapted score included dietary components only and ranged from 0 to 5 points.


**Supplementary Table S3. Plasma metabolites meeting BH-FDR <0.05 in both bridge paths**

| **NMR-derived measure** | **Specimen** | **a-path beta** | **FDR(a)** | **b-path HR (95% CI)** | **FDR(b)** |
| --- | --- | --- | --- | --- | --- |
| Saturated Fatty Acids to Total Fatty Acids percentage | Plasma | -0.126 | <0.001 | 1.193 (1.095, 1.300) | 0.009 |
| Linoleic Acid to Total Fatty Acids percentage | Plasma | 0.095 | <0.001 | 0.826 (0.752, 0.908) | 0.009 |
| Polyunsaturated Fatty Acids to Total Fatty Acids percentage | Plasma | 0.098 | <0.001 | 0.838 (0.765, 0.919) | 0.014 |

Note. All measures were quantified in plasma. Linoleic acid is a component of omega-6 polyunsaturated fatty acids; these proportional measures are correlated.


**Supplementary Table S4. Covariate availability audit**

| **Covariate** | **Available** | **Variable used in the model** | **Note** |
| --- | --- | --- | --- |
| Age | Yes | Age at dietary assessment | Included in the revised primary model |
| Sex | Yes | Sex | Included in the revised primary model |
| Ethnicity | Yes | Ethnicity | Included in the revised primary model |
| Education | Yes | Educational attainment | Included in the revised primary model |
| Household income | Yes | Household income | Included in the revised primary model |
| Smoking | Yes | Smoking status | Included in the revised primary model |
| Alcohol | Yes | Alcohol consumption | Included in the revised primary model |
| BMI | Yes | Body mass index | Included in the revised primary model |
| Physical activity | Yes | Physical activity (log-transformed MET-min/week) | Included in the revised primary model |
| Diabetes | Yes | Diabetes status | Included in the revised primary model |
| Total energy intake | Yes | Total energy intake | Included in the revised primary model |
| Townsend deprivation index | No |  | UKB Field 189 was not present in the local export; requires re-export |
| Assessment centre | No |  | Not present in the local export |

Note. Townsend deprivation index corresponds to UKB Field 189 and was absent from the local analytical export.


**.**

**Supplementary Table S5. Baseline comparison of included and excluded eligible participants**

| **Characteristic** | **Category** | **Included** | **Excluded** | **P value** | **SMD** |
| --- | --- | --- | --- | --- | --- |
| Age, years |  | 55.53 (7.86) | 56.08 (7.82) | 0.002 | -0.069 |
| Body mass index, kg/m2 |  | 26.64 (4.51) | 27.18 (5.12) | <0.001 | -0.111 |
| Physical activity, log MET-min/week |  | 7.25 (1.36) | 7.17 (1.44) | 0.011 | 0.059 |
| Total energy intake |  | 8709.82 (2185.44) | 8490.67 (2185.09) | <0.001 | 0.100 |
| WCRF/AICR dietary score |  | 2.56 (1.00) | 2.59 (1.00) | 0.284 | -0.033 |
| Valid dietary recalls, n |  | 2.96 (0.89) | 2.94 (0.90) | 0.444 | 0.015 |
| Sex | Female | 48,592 (52.9%) | 1,103 (57.0%) | <0.001 | 0.084 |
|  | Male | 43,325 (47.1%) | 831 (43.0%) |  |  |
| Ethnicity | White | 88,993 (96.8%) | 1,853 (95.8%) | 0.015 | 0.053 |
|  | Non-white or unknown | 2,924 (3.2%) | 81 (4.2%) |  |  |
| Education | University degree | 45,353 (49.3%) | 883 (45.7%) | 0.003 | 0.074 |
|  | Other qualification | 41,458 (45.1%) | 918 (47.5%) |  |  |
|  | No formal qualification | 4,919 (5.4%) | 129 (6.7%) |  |  |
|  | Unknown | 187 (0.2%) | 4 (0.2%) |  |  |
| Household income | Low | 29,919 (32.6%) | 687 (35.5%) | 0.005 | 0.070 |
|  | Middle | 24,765 (26.9%) | 539 (27.9%) |  |  |
|  | High | 30,419 (33.1%) | 577 (29.8%) |  |  |
|  | Unknown | 6,814 (7.4%) | 131 (6.8%) |  |  |
| Smoking status | Never | 52,861 (57.5%) | 1,123 (58.1%) | 0.929 | 0.012 |
|  | Previous | 32,466 (35.3%) | 675 (34.9%) |  |  |
|  | Current | 6,456 (7.0%) | 134 (6.9%) |  |  |
|  | Unknown | 134 (0.1%) | 2 (0.1%) |  |  |
| Alcohol use | Current | 86,842 (94.5%) | 1,813 (93.7%) | 0.177 | 0.031 |
|  | Non-current or unknown | 5,075 (5.5%) | 121 (6.3%) |  |  |
| Doctor-diagnosed diabetes | No or unknown | 88,559 (96.3%) | 1,852 (95.8%) | 0.194 | 0.030 |
|  | Yes | 3,358 (3.7%) | 82 (4.2%) |  |  |

Note. Continuous variables are mean (SD); categorical variables are n (%). SMD denotes standardized mean difference.


**Supplementary Table S6. Exploratory plasma metabolite module associations with incident lung cancer**

| **Data-driven module** | **HR (95% CI)** | **P value** | **FDR** |
| --- | --- | --- | --- |
| Fatty acid profile | 0.866 (0.785, 0.956) | 0.004 | 0.008 |
| Other metabolic signals | 0.893 (0.812, 0.982) | 0.019 | 0.025 |
| Lipoprotein-lipid profile | 0.913 (0.831, 1.003) | 0.059 | 0.059 |
| Energy metabolism | 1.141 (1.052, 1.238) | 0.001 | 0.006 |

Note. Modules were derived and tested in the same dataset and are presented as exploratory signal summaries.


**Supplementary Table S7. Selection-IPW specification and weight distribution**

| **Weight summary** | **Value** |
| --- | --- |
| mean | 1.0000 |
| sd | 0.0044 |
| min | 0.9923 |
| p01 | 0.9923 |
| p05 | 0.9939 |
| median | 0.9993 |
| p95 | 1.0083 |
| p99 | 1.0144 |
| max | 1.0144 |

Note. Variables in the selection model: Age at dietary assessment, Sex, Ethnicity, Educational attainment, Household income, Smoking status, Alcohol consumption, Body mass index, Physical activity (log-transformed MET-min/week), Diabetes status, Total energy intake, WCRF/AICR dietary adherence score, Number of valid 24-hour dietary recalls.


**Supplementary Table S8. Lagged, recall-count, selection-weighted, and competing-risk analyses**

| **Analysis** | **Exposure** | **Participants** | **Cases** | **HR/SHR (95% CI)** | **P value** |
| --- | --- | --- | --- | --- | --- |
| Exclude first 2 years | WCRF/AICR score | 89,199 | 391 | 0.968 (0.871, 1.076) | 0.545 |
| Exclude first 2 years | Plasma metabolomic signature | 89,199 | 391 | 0.802 (0.737, 0.873) | <0.001 |
| Exclude first 2 years | Plasma bridge score | 89,199 | 391 | 0.947 (0.850, 1.055) | 0.323 |
| Exclude first 3 years | WCRF/AICR score | 87,784 | 342 | 0.980 (0.876, 1.098) | 0.730 |
| Exclude first 3 years | Plasma metabolomic signature | 87,784 | 342 | 0.800 (0.729, 0.879) | <0.001 |
| Exclude first 3 years | Plasma bridge score | 87,784 | 342 | 0.951 (0.847, 1.068) | 0.398 |
| Exclude first 5 years | WCRF/AICR score | 84,693 | 264 | 1.003 (0.882, 1.140) | 0.966 |
| Exclude first 5 years | Plasma metabolomic signature | 84,693 | 264 | 0.799 (0.718, 0.889) | <0.001 |
| Exclude first 5 years | Plasma bridge score | 84,693 | 264 | 0.927 (0.813, 1.057) | 0.260 |
| At least 2 valid dietary recalls | WCRF/AICR score | 91,917 | 470 | 0.989 (0.898, 1.089) | 0.821 |
| At least 2 valid dietary recalls | Plasma metabolomic signature | 91,917 | 470 | 0.816 (0.752, 0.885) | <0.001 |
| At least 2 valid dietary recalls | Plasma bridge score | 91,917 | 470 | 0.907 (0.823, 1.000) | 0.051 |
| Selection-IPW Cox | WCRF/AICR score | 91,917 | 470 | 0.989 (0.895, 1.093) | 0.829 |
| Selection-IPW Cox | Plasma metabolomic signature | 91,917 | 470 | 0.816 (0.763, 0.873) | <0.001 |
| Selection-IPW Cox | Plasma bridge score | 91,917 | 470 | 0.908 (0.819, 1.005) | 0.064 |

Note. Selection-IPW weights were truncated at the 1st and 99th percentiles. Fine–Gray estimates are subdistribution hazard ratios.
